# Pituitary Morphology and Function in 43 Children with Central Diabetes Insipidus

**DOI:** 10.1155/2016/6365830

**Published:** 2016-03-29

**Authors:** Wendong Liu, Limin Wang, Minghua Liu, Guimei Li

**Affiliations:** ^1^Department of Pediatrics, Shandong Provincial Hospital Affiliated to Shandong University, Jinan 250021, China; ^2^Department of Pediatrics, Qingdao Municipal Hospital Affiliated to Qingdao University, Qingdao 266011, China; ^3^Department of Child Health Care, Shandong Maternal and Child Health Care Hospital, Jinan 250021, China

## Abstract

*Objective*. In pediatric central diabetes insipidus (CDI), etiology diagnosis and pituitary function monitoring are usually delayed. This study aimed to illustrate the importance of regular follow-up and pituitary function monitoring in pediatric CDI.* Methods*. The clinical, hormonal, and neuroradiological characteristics of children with CDI at diagnosis and during 1.5–2-year follow-up were collected and analyzed.* Results*. The study included 43 CDI patients. The mean interval between initial manifestation and diagnosis was 22.29 ± 3.67 months (range: 2–108 months). The most common complaint was polyuria/polydipsia. Causes included Langerhans cell histiocytosis, germinoma, and craniopharyngioma in 2, 5, and 4 patients; the remaining were idiopathic. No significant changes were found during the 1.5–2 years after CDI diagnosis. Twenty-three of the 43 cases (53.5%) had ≥1 anterior pituitary hormone deficiency. Isolated growth hormone deficiency was the most frequent abnormality (37.5%) and was not associated with pituitary stalk diameter. Multiple pituitary hormone deficiencies were found in 8 cases with pituitary stalk diameter > 4.5 mm.* Conclusion*. Diagnosis of CDI is usually delayed. CDI with a pituitary stalk diameter > 4.5 mm carries a higher risk of multiple pituitary hormone deficiencies. Long-term MRI and pituitary function follow-ups are necessary for children with idiopathic CDI.

## 1. Introduction

Central diabetes insipidus (CDI) results from deficiency synthesis, release of arginine vasopressin (AVP), or both. Polyuria and polydipsia are important clues to the diagnosis; nonetheless, approximately 40% of the patients have symptoms other than polyuria and polydipsia at presentation [1]. Manifestations can be affected by age, cause, or other dysfunctions. Infants may have fever, vomiting, or failure to thrive rather than polyuria and polydipsia. CDI may be a consequence of surgery or a component of multiple pituitary hormone deficiencies (MPHDs). Polyuria can be masked by adrenocorticotrophic hormone (ACTH) deficiency [2]. Water deprivation tests and a DDAVP (1-desamino-8-D-arginine-vasopressin) trial remain the diagnostic gold standard of CDI, often confirmed by direct measurements of serum AVP and copeptin [3–5].

The etiology of childhood CDI is heterogeneous. It can be familial (i.e., a genetic defect of AVP synthesis) or secondary to diseases that affect the hypothalamic-pituitary system, such as Langerhans cell histiocytosis (LCH), germinoma, craniopharyngioma, trauma related to surgery or accident, or cranial malformations. Hypothalamic magnetic resonance imaging (MRI) is necessary because it not only may lead to a diagnosis of CDI by lack of posterior pituitary bright spot on T1-weighted imaging, but also can reveal intracranial tumor or LCH with a thickened pituitary stalk. However, 20–50% of cases are considered idiopathic [1], either with normal or with thickened pituitary stalk. There may be a delay in the diagnosis of pituitary hormone deficiency, isolated growth hormone deficiency, or MPHD. However, the delay in diagnosis of diabetes insipidus did not affect survival rates significantly [6]. Furthermore, the underlying cause of pituitary stalk thickening associated with MPHD in idiopathic CDI is not completely understood.

To date, diagnosis and differential diagnosis of childhood CDI in China are daunting, with lack of systematic follow-up. In our study, we analyzed the clinical, hormonal, and neuroradiological data at diagnosis and follow-up to illustrate the importance of regular follow-up and monitoring of pituitary function.

## 2. Materials and Methods

### 2.1. Subjects

From 2012 to 2014, 43 patients (23 boys and 20 girls) ranging in age from 1.9 to 11.5 y were diagnosed with CDI at the Pediatric Endocrinology Department of Shandong Provincial Hospital. A family history, chief complaints, physical examinations, and laboratory and radiological evaluation were obtained at presentation. Diagnosis of CDI was based on a history of polyuria and polydipsia (urine volume > 1500 mL/kg/d at birth, 100–110 mL/kg/d at 2 y, and 40–50 mL/kg/d after 2 y [7]), with a consistent urine osmolality < 300 mOsm/kg in the 24 h urine specimen. All patients underwent an in-patient water deprivation test and then a desmopressin trial. Patients were treated with oral desmopressin acetate after confirmation of CDI, 2 or 3 times daily.

### 2.2. Water Deprivation Test and DDAVP Trial

The water deprivation test lasted 4 to 7 hours, in accordance with the standard protocol previously reported [9]. Blood pressure, pulse rate, and weight were monitored at baseline and hourly during the test. Urine samples were also collected hourly for the measurement of urine osmolality and specific gravity. Blood samples for measurement of serum sodium and plasma osmolality were obtained at the beginning and end of the water deprivation test.

After the water deprivation test, DDAVP was administrated by subcutaneous injection (0.1 *μ*g/kg, maximum 4 *μ*g) as a trial. Urine was collected hourly for osmolality and specific gravity. Serum sodium and osmolality were measured again 2 hours after DDAVP administration.

Complete CDI was defined as urine osmolality < 300 mOsm/kg after the water deprivation test with a >50% increase in response to DDAVP. Partial CDI and partial nephrogenic diabetes insipidus and defined as maximum urine osmolality between 300 and 800 mOsm/kg during the water deprivation test and <50% increase in response to DDAVP. Complete CDI, partial CDI, and partial nephrogenic diabetes insipidus were distinguished by abolishment of thirst, polyuria, and polydipsia, without development of hyponatremia, in response to a therapeutic trial with a standard dose of desmopressin [10].

### 2.3. Anterior Pituitary Function

Growth hormone deficiency was confirmed by provocative tests in all patients. Blood samples to measure growth hormone levels were obtained at baseline and at 30, 60, 90, and 120 min after the administration of arginine (0.5 g/kg, given intravenously over a period of 30 min) and levodopa (10 mg/kg, maximum 500 mg, given orally). Growth hormone deficiency was defined as growth hormone peak level < 10 ng/mL combined with a subnormal IG-1 (insulin-like growth factor 1) level for chronological age.

Thyroid-stimulating hormone (TSH) deficiency was defined as free thyroxine (FT4) < 12.0 pmol/L with a low or low-to-normal serum concentration of TSH.

Plasma ACTH and serum cortisol were measured in the morning (08:00 a.m.). ACTH deficiency was defined by either a morning serum cortisol concentration < 138 nmol/L or a peak serum cortisol concentration < 550 nmol/L during insulin-induced hypoglycemia, in concert with an inappropriately low plasma ACTH concentration.

Follicle-stimulating hormone/luteinizing hormone (FSH/LH) deficiency was diagnosed in 2 patients who had absent/delayed pubertal development. Serum FSH and LH were measured at 0 minutes and 30, 60, and 90 min after administration of gonadotropin-releasing hormone. Low serum testosterone in boys or estradiol in girls, combined with a blunted LH/FSH response to gonadotropin-releasing hormone, was diagnostic for FSH/LH deficiency.

### 2.4. MRI

All patients underwent hypothalamic-pituitary MRI scans, without and then with contrast agent, using a 3.0 T magnetic resonance scanning machine (Siemens & Co., Germany), in accordance with standard protocol (3.0 mm slice thickness, coronal and sagittal orientation, 256 × 256 matrix size, T1-weighted imaging without and then with contrast agent). The contrast agent was intravenous gadolinium-diethylenetriamine pentaacetic acid (Gd-DTPA), 0.1 mmol/kg.

Images were interpreted by 2 independent observers without prior knowledge of the patients, noting posterior pituitary bright spot, pituitary height, and thickness of the pituitary stalk. Pituitary height was compared to the normal value for age, gender, and development [11]. A thickened stalk was defined as a diameter > 3 mm in at least one of its 3 portions (proximal, median, and distal) [12].

The thickness of the pituitary stalk was characterized as mild (3.0–4.5 mm), moderate (4.6–6.5 mm), or severe (>6.5 mm) [1]. The height of the pituitary gland was compared to the gender and age reference established by Argyropoulou et al. [8].

Based on the hypothalamic-pituitary MRI, patients were considered TPS^−^ or TPS^+^ (diameter of pituitary stalk ≤ 3 mm or >3 mm, resp.).

### 2.5. Other Studies

To rule out LCH, 27 patients with thickened pituitary stalk underwent skeletal surveys, lung computed tomography (CT) scan, whole-body nuclear medicine bone scanning, and skin histopathological test. To detect secretary germinoma, alpha-fetoprotein (AFP) and *β*-human chorionic gonadotropin (*β*-HCG) levels in the blood and spinal fluid were also analyzed.

### 2.6. Follow-Up

During a 1.5-to-2 y follow-up, patients with idiopathic conditions were seen at 3–6-month intervals and given physical examination and tested for anterior and posterior pituitary function and also tumor markers (serum AFP and *β*-HCG). Hypothalamic MRI was repeated every 6–12 months. In those patients with a confirmed etiology, follow-up was conducted by phone or clinic.

### 2.7. Statistical Analysis

IBM SPSS Statistics ver. 20.0 (SPSS, Armonk, NY, USA) software was used for all statistical analyses. Student's unpaired *t*-test was performed for univariate comparisons between groups. Pearson's chi-squared test and Fisher's exact test were applied to compare proportions between groups. Statistical significance was set at *P* < 0.05.

## 3. Results

### 3.1. Baseline Clinical Data

There were 16 and 27 patients in the TPS^−^ and TPS^+^ groups, respectively. Of these 43 patients (23 boys, gender ratio: 1.15), the median age at CDI diagnosis was 7.47 ± 0.49 y (range: 1.9–11.5 y). The mean interval between the initial manifestation and diagnosis was 22.29 ± 3.67 (range: 2–108 months). There were no significant differences between the 2 groups (Table 1).

The most common symptoms or signs were polyuria/polydipsia (*n* = 39, 90.6%), nocturia (*n* = 18, 41.9%), short stature (*n* = 17, 39.5%), anorexia (*n* = 10, 23.3%), headache (*n* = 3, 6.9%), mental retardation (*n* = 2, 4.7%), emesis (*n* = 1, 2.3%), and rash (*n* = 1). There were 5 patients with a normal pituitary stalk and 12 with abnormal pituitary stalk accompanied by short stature, with heights of standard deviation of −3.48 ± 0.45 and −3.13 ± 0.29, respectively.

Two patients with mental retardation had comorbidity including hydronephrosis and ureterectasia; their intervals between presentation and diagnoses were 72 months and 108 months. Both had a poor response to DDAVP therapy.

None of the subjects had a familial etiology, nor a history of surgery, infection, or trauma. All TPS^−^ cases were idiopathic, whereas 11 TPS^+^ patients were etiologically diagnosed with germinoma (*n* = 5), craniopharyngioma (*n* = 4), and LCH (*n* = 2; Table 2). Short stature, defined as individual height > 2 standard deviations below the corresponding mean height for a given age, gender, and population, was present in 10 patients, except one patient with LCH.

### 3.2. Posterior Pituitary Function

Urine volume of the 43 patients was 202.7 ± 9.83 mL/kg/day. Initial urine osmolality was 128.6 ± 6.8 mOsm/kg and serum osmolality was 286.2 ± 1.6 mOsm/kg. The ratio of urine osmolality to serum osmolality was <1.0 and increased significantly to 179.1 ± 0.5 mOsm/kg and 297.5 ± 1.9 mOsm/kg, respectively, after water deprivation (*P* < 0.01). In response to DDAVP, urine osmolality increased to 492 ± 18.6 mOsm/kg, with a mean increment of 202.4 ± 17.51% (range: 39–450%). One patient received a diagnosis of partial CDI (urine osmolality increased by 39%) with a standard dose of desmopressin, and 42 patients had complete CDI. There were no differences between the two groups (Table 2).

### 3.3. Neuroimaging Findings

The posterior pituitary bright spot was absent on T1-weighted MRI in all 43 patients (100%). Of the 27 patients with abnormal pituitary stalk, hypothalamic-pituitary MRI revealed suprasellar mass in 9: germinoma (*n* = 5), supported by elevated serum CSF/*β*-HCG level, and craniopharyngioma (*n* = 4) based on histological examination after surgery. Two children had LCH with thickened pituitary stalk, confirmed histopathologically in skin or skull lesion.

Craniopharyngioma patients (*n* = 4) had an invisible pituitary stalk and anterior pituitary gland. In 2 of 3 germinoma patients with severe thickened pituitary stalk, the anterior pituitary was not seen on MRI, whereas the other had a reduced anterior pituitary size. Anterior pituitary glands in the 3 patients with moderately thickened pituitary stalk (1 with LCH and 2 with germinoma) were smaller than normal. Seventeen patients (1 with LCH and 16 with idiopathic cases) showed mildly thickened pituitary stalk, 3 of whom had a smaller anterior pituitary gland than normal. Of the 16 patients with normal pituitary stalk, 4 had hypoplastic anterior pituitaries.

In summary, anterior pituitary size was reduced in patients with normal or abnormal pituitary stalk and was undetected by MRI in patients with severely thickened and invisible pituitary stalk.

### 3.4. Anterior Pituitary Function

Anterior pituitary function was assessed in 43 patients; 23 (53.5%) had at least 1 anterior pituitary hormone deficiency. Isolated growth hormone deficiency was the most frequent abnormality (*n* = 15, 37.5%) and was not associated with pituitary stalk size.

Among the 16 patients with normal pituitary stalk, 6 (37.5%) had isolated growth hormone deficiency, 4 with small and 2 with normal anterior pituitary.

Among the 17 patients with mildly thickened pituitary stalk, 6 showed isolated growth hormone deficiency, 3 of whom had decreased size of anterior pituitary. Anterior pituitary hormone function was normal in 11 patients. MPHD was found in 2 patients with a moderately thickened pituitary stalk, and 2 of 3 patients with severely thickened pituitary stalk developed growth hormone deficiency and TSH deficiency. All 4 patients with an invisible pituitary stalk had MPHD (Table 3). In addition, 1 patient with germinoma and 1 patient with craniopharyngioma had elevated PRL levels (29.03 ng/mL and 58.54 ng/mL). Our study found an association between thickness of the pituitary stalk and the likelihood of MPHD (*P* < 0.05).

### 3.5. Follow-Up

Five patients with germinoma were treated with chemotherapy and then radiotherapy. Four patients with craniopharyngioma underwent surgical excision and pituitary stalk was undetectable on MRI. All patients with tumor developed at least 3 pituitary hormone deficiencies. Two patients with LCH are monitored regularly, and one was found to have isolated growth hormone deficiency. One craniopharyngioma patient relapsed 1.5 years after surgery.

One idiopathic patient with a mildly thickened pituitary stalk (3.4 mm) was normal one year after diagnosis but still required DDAVP therapy. The pituitary stalk of one patient increased from 3.9 mm to 4.0 mm after 6 months. Others did not have any significant change and are still under follow-up.

## 4. Discussion

We analyzed the clinical, biochemical, neuroradiological, and hormonal results from pediatric patients with CDI.

Polyuria and polydipsia are the most common manifestations of CDI. However, some patients were seen by the doctor because of slow growth. In addition, polyuria and polydipsia can be masked by ACTH deficiency [2]. A previous study reported that 40% of CDI patients had symptoms other than polyuria/polydipsia at presentation [1]. In the present study, 12 patients (27.9%) presented with short stature and had growth hormone deficiency, and another 5 patients presented with polyuria/polydipsia accompanied by short stature. Short stature and growth hormone deficiency were not associated with etiology, which is consistent with previous studies [13]. However, short stature can be a predictor of growth hormone deficiency. In addition, other symptoms or signs such as vomiting and headache might be related to intracranial tumors, and rash might be a predictor of LCH.

In the present study, the mean age at diagnosis was 7.47 ± 0.49 years, in accord with previous reports [14], widely ranging from 1.9 to 11.5 years. The time interval between onset and diagnosis varied by wide range. However, for 76.7%, the interval was more than 12 months; most children's heights were below the 50th percentile and the body mass index was low. Two patients with longer intervals between onset and diagnosis (108 and 72 months, resp.) developed nephritic complications. Delay in diagnosis can affect life quality due to complications.

Polydipsia and polyuria exceeding 5 mL/kg/hour with urine specific gravity less than 1.01, plasma sodium level above 145 mmol/L, and dehydration are the hallmarks of diabetes insipidus in infants and children [15]. In the present study, the mean urine volume over 24 hours was 202.7 ± 9.83 mL/kg, approximately equal to 8.4 mL/kg/hour, much higher than 5 mL/kg/hour. Baseline levels of urine osmolality were lower than 300 mOsm/kg, and urine specific gravity was below 1.010. However, the average plasma sodium was 145.4 ± 1.4 mmol/L, which is not consistent with a previous hypernatremia report.

As a type of diabetes insipidus, it is important to differentiate CDI from nephrogenic diabetes insipidus and primary polydipsia, because inappropriate treatment can result in severe complications. Direct measurement of serum AVP is recommended for diagnosis [3, 4], but it has not been accepted due to several shortcomings [1, 16–21]. In contrast, copeptin, the C-terminal glycoprotein moiety of pro-AVP, has been suggested as a stable surrogate marker of AVP secretion to improve the differential diagnosis of diabetes insipidus [16]. A recent prospective multicenter study showed significant lower copeptin levels (both baseline and osmolality stimulated) in CDI cases [5]. However, we did not test AVP and copeptin when our patients were diagnosed, due to limitations.

The water deprivation test and DDAVP trial still are the gold standard for diagnosis/differential diagnosis of CDI. But the interpretation is more and more challenging. For example, we confirmed 42 complete CDI and 1 partial CDI using the protocol in our study, but this would have been 39 and 4, respectively, if we defined complete CDI as a ratio of urinary osmolality to plasma osmolality of ≤1.0 and partial CDI as >1.0 [1]. Only four patients with baseline urinary osmolality < 300 mOsm/kg showed an increase to >750 mOsm/kg after water deprivation, and this may be considered partial CDI if we used another protocol [22]. Although there would be different proportions of partial CDI and complete CDI given different interpretations, there was no correlation with ideology. All the patients responded to DDAVP treatment. This suggests that a therapeutic trial with the standard dose of desmopressin is important to differentiate partial CDI, partial nephrogenic diabetes insipidus, and primary polydipsia, if measurements of AVP and copeptin are limited.

MRI is recommended as soon as CDI is diagnosed. One common finding in MRI is the absence of a physiological posterior bright spot (PBS) on sagittal T1-weighted imaging. Some earlier reports showed that absence of a PBS is not specific for CDI diagnosis, because individual CDI cases with persistent PBS have been reported [23, 24]. However, the PBS is diminished or absent in both CDI and nephrogenic diabetes insipidus [25–27], and it is always absent when diabetes insipidus was a presenting symptom [13, 28]. All the cases in our study lack the PBS (100%).

A thickened pituitary stalk, isolated or with a mass, is another common finding on MRI, in 50–60% of the children. LCH, germinoma, and craniopharyngioma are the most common causes of thickened pituitary stalk, but neither the pattern nor the thickness of the pituitary stalk correlated with clinical features. Radiological features of craniopharyngioma are typical and easy to differentiate from LCH and germinoma [2]. It was difficult to distinguish on MRI between LCH and germinoma because LCH can be single system that only involves the hypothalamic-pituitary system; tumor markers such as AFP and *β*-HCG in the blood and central spinal fluid can be negative in the early stage of the tumor; and LCH and germinoma could share the same findings in some cases when CDI was diagnosed.

A biopsy of the pituitary stalk can provide a definitive diagnosis. However, physicians in China are conservative on biopsy, due to the risk of surgery and because of the parents. In our study, 2 of the 5 germinoma patients were diagnosed through elevated plasma *β*-HCG levels. One LCH patient had a skin rash, while the other 3 germinoma patients and 1 LCH patient were diagnosed by the good response to the trial therapeutic treatment (Table 4). Four craniopharyngioma patients had a typical pattern on MRI, which was confirmed by surgery and pathology.

The height of the pituitary gland on MRI can be normal, low, or high. Maghnie et al. [1] suggested that an associated large antehypophysis and a thickened stalk are highly suspicious of a germinal tumor. However, germinoma cases in our study had a small or invisible pituitary gland.

Dysfunction of the anterior pituitary can be determined at diagnosis and during the follow-up period of CDI. In the present study, 53.5% of the patients had anterior pituitary hormone deficiency when CDI was diagnosed, which was lower than the 80% reported by Liu et al. [29]. Most of these patients had growth hormone deficiency, not related to etiology.

In our study here, the probability of developing MPHD was associated with thickness of the pituitary stalk. In addition, our study found higher prevalence of MPHD in cases with intracranial tumor compared with LCH. Marchand et al. [30] found that the number of anterior pituitary deficiencies seemed to be dependent on the size of the initial lesion. In our patients, 2 LCH patients, there was no MPHD, perhaps because the sizes of the neoplasms were less than 6.5 cm. However, long-term monitoring is needed, because this can develop years after CDI [30, 31].

The natural history of CDI in terms of etiology is hard to determine. Clinical, radiological, and endocrinological investigations must be performed at diagnosis and follow-up. During follow-ups that ranged from 4.1 to 14.3 years of 43 idiopathic CDI patients (mean age: 7.4 y), 3 patients had LCH and one patient developed Hodgkin's lymphoma 8.5 to 10 years after CDI onset [32]. Although the follow-up times of our present cases were not long enough, they are still being monitored. An appropriate therapeutic trial may be useful in patients with a persistent uncertain diagnosis; however, it should be conducted with close supervision.

In this study, we have demonstrated the limitations in the diagnosis and follow-up of childhood CDI in China. These limitations are important to note, and the attention of parents and physicians given to the associated symptoms, signs, and diagnostic tests should be improved to prevent delay in diagnosis. As soon as there is a diagnosis of CDI, regular and long-term clinical follow-ups are required that include imaging and tests for tumor markers and pituitary function.

## Figures and Tables

**Table 1:** Baseline data of all patients with CDI.

		Total	TPS^−^	TPS^+^	*P*
Subjects, *n*		43	16	27	—

Male/female, *n*		23/20	12/4	11/16	—
Age at diagnosis, y		7.47 ± 0.49	6.58 ± 0.67	8.01 ± 0.66	0.16
Interval, months		22.29 ± 3.67	18.86 ± 5.99	24.32 ± 4.69	0.48

Short stature, *n*		17 (39.5%)	5 (31.3%)	12 (44.4%)	0.39
Height SDS		–3.23 ± 0.24	–3.48 ± 0.45	–0.13 ± 0.29	—
BMI, kg/m^2^		16.12 ± 0.29	15.98 ± 0.42	16.20 ± 0.39	0.7
Urine 24 h, mL/kg		202.7 ± 9.83	188.6 ± 12.99	211.0 ± 13.56	0.27

APD		23 (53.5%)	6 (37.5%)	17 (63%)	0.11
	IGHD	15	6 (37.5%)	9 (33.3%)	0.36
	MPHD	8	0	8 (29.6%)	0.02^*∗*^

GHD +1		5	0	5	—
GHD +2^*∗*^		1	0	1	—
GHD +3^*∗*^		2	0	2	—

Pituitary bright spot		Absent (100%)	Absent (100%)	Absent (100%)	—

AP	Normal	26 (60.5%)	12 (75%)	14 (51.9%)	0.13
	<Normal	11	4 (25%)	7 (25.9%)	0.95
	Not visible	6	0	6 (22.2%)	0.04^*∗*^

^*∗*^ *P* < 0.05.

AP: anterior pituitary; APD: anterior pituitary deficiency; GHD: growth hormone deficiency; IGHD: isolate growth hormone deficiency.

**Table 2:** Plasma and urine chemistry.

		Baseline	End of WD	End of DDAVP
Plasma Na^+^	Total	144.3 ± 0.9	150.0 ± 0.9	139.9 ± 0.8
	TPS^−^	145.4 ± 1.4	151.1 ± 1.7	137.9 ± 0.8
	TPS^+^	143.6 ± 1.1	149.4 ± 0.9	141.1 ± 1.0

Serum osmolality	Total	286.2 ± 1.6	297.5 ± 1.9	278.7 ± 1.3
	TPS^−^	287.5 ± 2.8	298.4 ± 3.8	275.4 ± 1.5
	TPS^+^	285.4 ± 1.9	297.0 ± 2.2	280.6 ± 1.8

Urinary osmolality	Total	128.6 ± 6.8	179.1 ± 0.5	492 ± 18.6
	TPS^−^	122.2 ± 14.1	187.2 ± 23.3	491.6 ± 29.9
	TPS^+^	132.4 ± 6.9	174.3 ± 9.8	492.2 ± 24.1

Urinary specific gravity		1.0 to 1.004	1.0 to 1.007	1.01 to 1.023

WD: water deprivation.

**Table 3:** Pituitary function of 27 patients with abnormal pituitary stalk^*∗*^.

		Anterior pituitary		MPHD
	Patients	Invisible	<Normal	
Mild	17	0	3	0
Moderate	3	0	3	2 (66.7%)
Severe	3	2	1	2 (66.7%)
Invisible	4	4	0	4 (100%)

^*∗*^Reported as *n* or *n* (%).

**Table 4:** Features of CDI patients.

Patient	Cause of CDI	Age, y^a^	Gender	PSD	AP, mm^b^	Signs and symptoms		PHD
1	Germinoma	9.2	F	17	I	Polyuria, polydipsia, short stature, and anorexia	Trial diagnostic radiation	GH + TSH

2	Germinoma	2.7	F	6.5	<N	Polyuria, polydipsia, and short stature	Trial diagnostic chemotherapy	GH + TSH

3	Germinoma	4.9	F	6.7	<N	Polyuria, polydipsia, short stature, and vomit	Plasma *β*-HCG ↑	GH + TSH + ACTH, PRL ↑

4	Germinoma	7.6	F	5.5	<N	Polyuria, polydipsia, short stature, and anorexia	Plasma *β*-HCG ↑	GH + TSH

5	Germinoma	9	M	6.7	I	Short stature, polyuria, and polydipsia	Trial diagnostic radiation	GH

6	Craniopharyngioma	11.5	F	I	I	Short stature and headache	Histological examination after surgery	GH + TSH + ACTH + GnRH

7	Craniopharyngioma	7.9	M	I	I	Short stature and headache	Histological examination after surgery	GH + TSH + ACTH + GnRH, PRL ↑

8	Craniopharyngioma	3	M	I	I	Short stature, polyuria, and polydipsia	Histological examination after surgery	GH + TSH

9	Craniopharyngioma	5.9	M	I	I	Short stature, polyuria, polydipsia, and headache	Histological examination after surgery	GH + TSH

10	LCH	10.6	F	5.5	<N	Polyuria and polydipsia	Trial diagnostic chemotherapy	No

11	LCH	3.5	F	3.9	N	Polyuria, polydipsia, short stature, and rash	Skin biopsy	GH

12–27	Idiopathic	<4.5^c^			<N^d^			GH^e^
		To 6.5^f^			<N^f^			GH^f^

^a^Age at diagnosis; ^b^N: normal, 1.1–5 y (4.0 ± 0.7 mm), 5.1–10 (4.5 ± 0.6 mm), and 10.1–15 (5.3 ± 0.8 mm) [8]; ^c^ *n* = 15; ^d^ *n* = 2/12; ^e^ *n* = 6; ^f^ *n* = 1.

AP: anterior pituitary; GH: growth hormone; I: invisible; N: normal; PHD: pituitary hormone deficiency; PSD: pituitary stalk diameter.
